# Empathy decline at older age?

**DOI:** 10.18632/aging.101467

**Published:** 2018-06-01

**Authors:** Claus Lamm, Federica Riva, Giorgia Silani

**Affiliations:** 1 Social, Cognitive and Affective Neuroscience Unit, Department of Basic Psychological Research and Research Methods, Faculty of Psychology, University of Vienna, Vienna, Austria; 2 Department of Applied Psychology: Health, Development, Enhancement and Intervention, Faculty of Psychology, University of Vienna, Vienna, Austria

**Keywords:** empathy, healthy aging, fMRI

Empathy is a cornerstone of human communication and social interaction. It allows us to not only *know* what someone else is feeling, but to experience and share another person’s emotions - as if we were partially feeling them ourselves ([1] for review). While precursors of empathy such as emotion contagion exist from early infancy, more complex empathic responses require neurodevelopmental processes that might not have reached full maturation until adulthood. On the other end of the age range, neurodegenerative disorders such as fronto-temporal dementia have been associated with a partial loss of empathic abilities. Much less is known, though, about whether empathy also shows a decline with healthy aging, and how this is associated with changes in the underlying neural circuitry. In a recent functional magnetic resonance imaging (fMRI) study [2], thus aimed to pinpoint age-related changes in the neural substrates of empathy, using a cross-sectional design comparing healthy adolescents, young adults and persons of older age (around 16, 25 and 63 years, on average). Their main finding was that while adolescents and young adults did not show significant differences in neural activation, participants of older age showed reduced neural responses in a key area for empathic responding, the anterior insular cortex. While being largely in line with previous research by other groups [3], several aspects of this new study are noteworthy. First, older adults differed from younger ones only with respect to their neural activation, but not in their empathic assessments. This indicates that neural data may be more sensitive than behavioral data to pick up first signs of a decrease in empathy. Second, the observed differences could not be explained by differences in the way participants experienced emotions themselves, as a control condition testing first-hand emotion processing did not yield any age-related differences. Third, the observed reduction in empathy-related activity was not due to age-related differences in Theory of Mind, a socio-cognitive skill that has been speculated to account for age-related problems in social behavior (for a review see [4]). This suggests that they are genuinely related to brain activation changes in the affective domain, and the ability to share others’ feelings rather than only to know about them. Importantly, the latter aspect receives decisive support by this being the first study which directly showed age-related empathy differences to occur in areas related to the first-hand processing of the emotions participants were empathizing with. Taken together, the findings of Riva et al. [2] along with similar research published beforehand may suggest a need for preventive measures tailored at the maintenance of empathic skills in older age. However, several aspects need to be clarified first before drawing such conclusions. Foremost, the experimental setup of this and most previous studies required participants of all ages to empathize with a young adult. This leaves open the question whether the decrease in empathy-related activations is universal, or only affects how older people understand young adults – an almost stereotypical conclusion to which certainly both older and younger adults would subscribe to. Moreover, it remains to be shown whether older age not only impacted on how participants represented the feelings of their counterparts, but whether this also would have an effect on social behavior and interaction – an aspect that was outside the focus of the study at hand. Finally, a clearer understanding of the specific neural mechanisms of the observed differences is needed. As a method, fMRI is certainly very useful when it comes to identify activation differences between certain groups or experimental conditions. However, it has its limitations when it comes to determine what possibly caused these differences. Several candidates exist in the present case. They range from more biological ones, such as neurodegenerative processes affecting the neural structure and thus functionality of the identified areas when processing social cues. Note though that the study did not find differences in grey matter volume, which however is a rather crude measure of neurodegeneration. A more behavioral cause could be a reduced exposure of older adults to social interaction, resulting in reduced functionality of the engaged brain areas due to lack of use rather than structure. Depending on which mechanisms are determined by future research, different types of interventions or preventive measures need to be pursued to prevent a possible decline of empathy with older age.
